# Changes in glycoprotein expression between primary breast tumour and synchronous lymph node metastases or asynchronous distant metastases

**DOI:** 10.1186/s12014-015-9084-7

**Published:** 2015-05-12

**Authors:** Emila Kurbasic, Martin Sjöström, Morten Krogh, Elin Folkesson, Dorthe Grabau, Karin Hansson, Lisa Rydén, Sofia Waldemarson, Peter James, Emma Niméus

**Affiliations:** Department of Immunotechnology, House 406, Medicon Village, SE-223 81 Lund, Sweden; Division of Oncology and Pathology, Department of Clinical Sciences Lund, Lund University, Medicon Village, SE-223 81 Lund, Sweden; Amber Biosciences AB, Skrivarevägen 9, SE-22657 Lund, Sweden; Department of Pathology, Skåne University Hospital, SE-22185 Lund, Sweden; Department of Surgery, Clinical sciences, Lund University, SE-22185 Lund, Sweden; Department of Surgery, Skåne University Hospital, SE-22185 Lund, Sweden

**Keywords:** Breast cancer, Lymph node metastases, Distant metastases, Biomarkers, Glycopeptide capture, Secreted proteins, Membrane proteins, Mass spectrometry

## Abstract

**Background:**

Breast cancer is a very heterogeneous disease and some patients are cured by the surgical removal of the primary tumour whilst other patients suffer from metastasis and spreading of the disease, despite adjuvant therapy. A number of prognostic and treatment predictive factors have been identified such as tumour size, oestrogen (ER) and progesterone (PgR) receptor status, human epidermal growth factor receptor type 2 (HER2) status, histological grade, Ki67 and age. Lymph node involvement is also assessed during surgery to determine if the tumour has spread which requires dissection of the axilla and adjuvant treatment. The prognostic and treatment predictive factors assessing the nature of the tumour are all routinely based on the status of the primary tumour.

**Results:**

We have analysed a unique tumour set of fourteen primary breast cancer tumours with matched synchronous axillary lymph node metastases and a set of nine primary tumours with, later developed, matched distant metastases from different sites in the body. We used a pairwise tumour analysis (from the same individual) since the difference between the same tumour-type in different patients was greater. Glycopeptide capture was used in this study to selectively isolate and quantify N-linked glycopeptides from tumours mixtures and the captured glycopeptides were subjected to label-free quantitative tandem mass spectrometry analysis. Differentially expressed proteins between primary tumours and matched lymph node metastasis and distant metastasis were identified. Two of the top hits, ATPIF1 and tubulin β-chain were validated by immunohistochemistry to be differentially regulated.

**Conclusions:**

We show that the expression of a large number of glycosylated proteins change between primary tumours and matched lymph node metastases and distant metastases, confirming that cancer cells undergo a molecular transformation during the spread to a secondary site. The proteins are part of important pathways such as cell adhesion, migration pathways and immune response giving insight into molecular changes needed for the tumour to spread. The large difference between primary tumours and lymph node and distant metastases also suggest that treatment should be based on the phenotype of the lymph node and distant metastases.

**Electronic supplementary material:**

The online version of this article (doi:10.1186/s12014-015-9084-7) contains supplementary material, which is available to authorized users.

## Background

Breast cancer is the most common cancer among women and approximately 1500 women in Sweden die from the disease every year. Although the prognosis has improved during the last decade, there is still today no curable treatment for patients with a spread to distant sites. In order to prevent the development of distant spread, a combination of local and systemic treatment is used, and the choice is mainly based on the characteristics of the primary tumour [1]. The presence of lymph node metastases is investigated with sentinel node or a combination of sentinel node and axillary dissection, operations performed at the same time as the primary surgery.

Distinct genes and pathways within a given subtype of breast cancer, that appear to play an important prognostic role with regard to metastatic behaviour, have been identified such as the S100 and EpCAM families [2]. Some micro-metastatic foci will ultimately form macroscopic tumours due to their ability to recruit the necessary stroma and vasculature. They somehow avoid detection by the immune system and elimination [3]. Tumour development is essentially Darwinian, in that any of a number of molecular pathways that have been selected for in a specific tumour cell can contribute to the ‘successful’ metastatic tumour [4]. Moreover, this heterogeneity is dynamic, as selective pressures change (that is, in the new environment encountered by a metastatic cell in a secondary tissue site). Metastasis to axillary lymph nodes have been shown to be the most important independent prognostic factor and thus a risk factor for development of distant metastases in breast cancer [5,6].

Relatively few studies have investigated the changes occurring during the evolution of metastasis in comparison to the primary tumour. Prognostic factors and molecular subtypes have been investigated, comparing the primary tumour and synchronous lymph node metastases and comparing the primary tumour to both synchronous lymph node metastases and asynchronous distant metastases [7,8]. The shift in prognostic factors and molecular subtype was shown to be of clinical importance and indicated that biomarkers in the synchronous and asynchronous lymph nodes and distant metastases may affect the choice of treatment [9,10].

Another recent report [11] demonstrated how next generation sequencing can be used to determine the occurrence of somatic mutations occurring during the development and progression of lobular breast cancer. Using DNA and RNA re-sequencing, 32 somatic non-synonymous mutations in a metastatic tumour were found, 19 of which were not present in the primary lesion. In addition, RNA sequencing detected two new RNA editing events that recode the amino acid sequences of two proteins, SRP9 and COG3.

Mass spectrometric analysis has also been performed using 2-DE in two studies with matched primary breast tumour and lymph node metastases. In one of the studies, also using CGH array, there were similarities between the primary tumour and the lymph node metastases although distinct changes in the levels of individual proteins were shown (13 pairs), [12] whereas the other study showed larger differences in protein expression (5 pairs) [13]. Matching of the primary tumour and later developed distant recurrences is rare since the relapses normally are not operated. Dumont et al. matched one patient with the primary tumour and a bone metastasis and found significantly regulated surface proteins [14].

In the present study, we collected matched primary tumours with synchronous lymph node metastases and asynchronous distant metastases. This unique tumour set was then enriched by a glycocapture approach [15] to enable a proteomic analysis of an interesting sub-proteome. Glycoproteins are normally present in the plasma membrane, the extracellular-matrix or are secreted to the extra cellular space, and may thus be found in the blood. They are also known to mediate cellular processes important to cancer progression such as cell-to-cell signalling, growth, differentiation and migration [16,17]. The glycoprotein enriched samples were then analysed with liquid chromatography tandem mass spectrometry (LC-MS/MS) and were quantified with a label-free approach to find differentially regulated proteins, which can give a molecular insight in how the primary tumours evolves to metastatic potential. The differentially regulated proteins may also be exploited as biomarkers or treatment targets. Two of the top regulated proteins were validated with immunohistochemistry in a separate patient cohort.

## Results and discussion

### Experimental design

**Figure 1:**
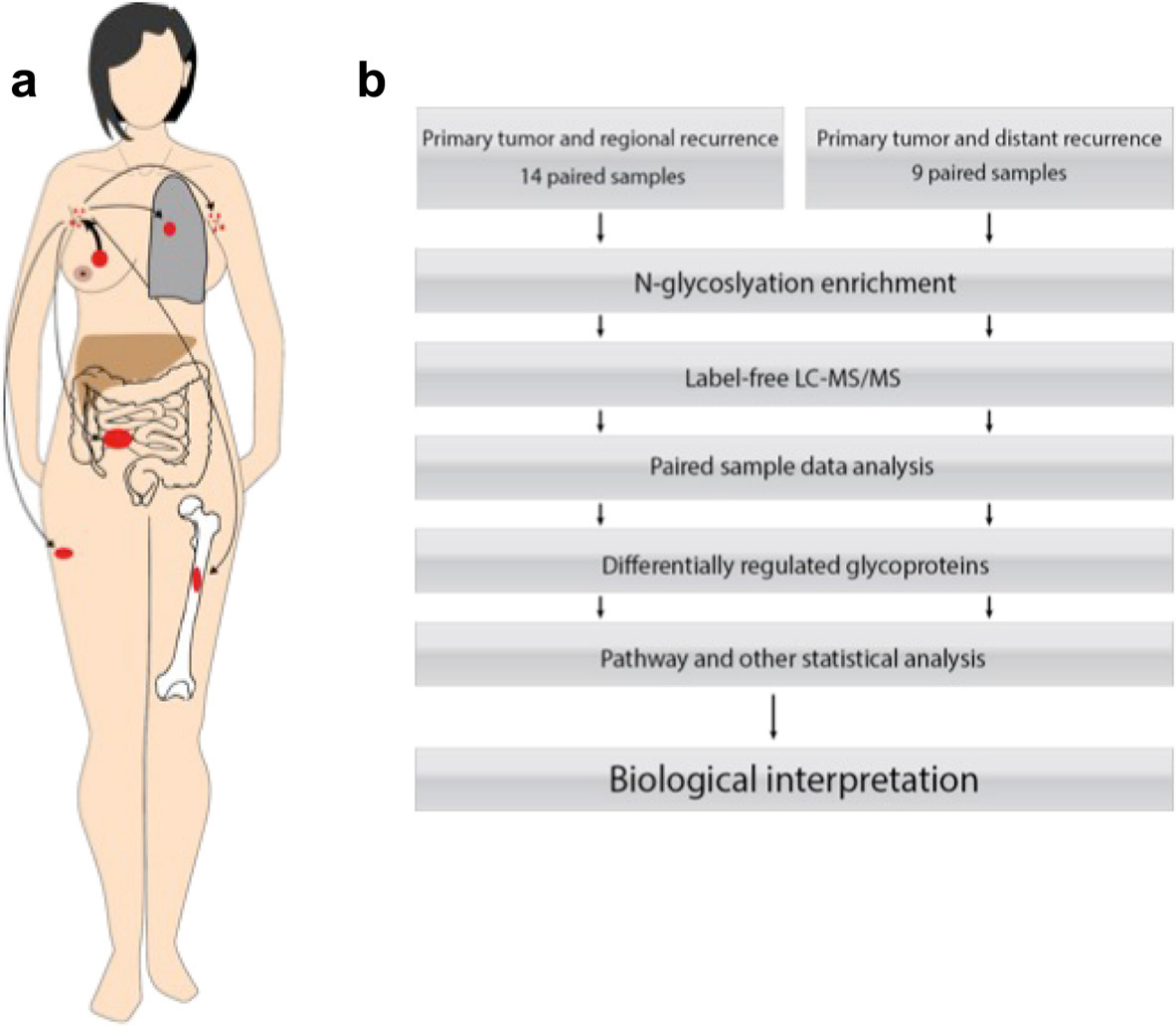
**(a)** A representation of cancer spread from primary tumour to synchronous lymph node metastases or asynchronous distant metastases. This figure shows a schematic representation of the spread of cancer from the primary tumour to the lymph nodes and distant sites for the samples in this study, which are lung, ipsilateral and contralateral axillary lymph nodes, bone and skin and in the greater oment. **(b)** Experimental design overview.

Samples were collected from 14 patients with tissue from the primary tumour and synchronous lymph node metastases (LNM) and 9 patients with tissue from a primary tumour and the distant metastasis (DM). LNM was defined as cancer involved lymph nodes in the ipsilateral axilla and DM as other sites than the ipsilateral breast or axilla. Two patients in the DM group had two samples, one had two skin metastases and one had metastases to the lung and to the ipsilateral breast (local recurrence). Most often distant metastases are not removed so the number is limited; however it is the largest proteomic study of its kind to be presented, to the best of our knowledge. The patient characteristics are given in Table 1, and the sites of primary tumours, lymph node metastases and distant metastases in this study are schematically shown in Figure 1(a).

**Table 1:** **Patient characteristics**

	**Lymph node metastasis**	**Distant metastasis**
Number of patients	14	9
with 2 samples	14	7
with 3 samples	0	2
Age, mean, range (years)	54, 25-79	53, 36-68
Site of metastasis		
Axilla	14	
Abdomen		3
Lung		2
Skeleton		2
Skin		2
Ipsilateral breast		1
Contralateral axilla		1
Tumour size^a^, mean range (mm)	35, 10-110	19, 13-28
Missing	0	2
ER^a^		
Positive	8	6
Negative	6	3
ER^b^		
Positive	7	4
Negative	5	4^c^
Missing	2	3
Adjuvant treatment^d^		
Radiotherapy	78%	22%
Endocrine therapy	64%	22%
Chemotherapy	43%	11%
Neoadjuvant treatment^e^		
Chemotherapy	14%	0%

^a^Of the primary tumour.

^b^Of the metastasis.

^c^One patient had an ER+ primary tumour and an ER- distant metastasis.

^d^After primary surgery.

^e^Before primary surgery.

Breast cancer is a heterogeneous disease at the pathological and histological level [18], and falls into at least 5 molecular subtypes (Luminal A and B; Basal; Her2 and normal), [19] and possible a sixth less abundant type, (low Claudin) [20] which greatly affect the prognosis [21]. Given the heterogeneity, we decided to take a paired approach in which both tumours come from the same patient. This allows a direct comparison of how the primary tumour has evolved to the lymph node and distant metastases. The experimental flow is shown in Figure 1(b).

We chose to focus on changes in glycoprotein expression during tumour progression. This allows us to focus on proteins from the plasma membrane, extracellular matrix and secreted proteins that are known to be important for cancer progression.

After glycopeptide enrichment, label-free LC-MS/MS was performed to identify and quantify the glycopeptides. Statistical and pathway analysis was performed to analyse the differences between primary tumours and the matched metastases. Finally, we verified the accuracy of LC-MS/MS with immunohistochemistry and validated two proteins to be differentially regulated.

### Variation in glycoprotein expression between the primary tumour and metastases

**Figure 2:**
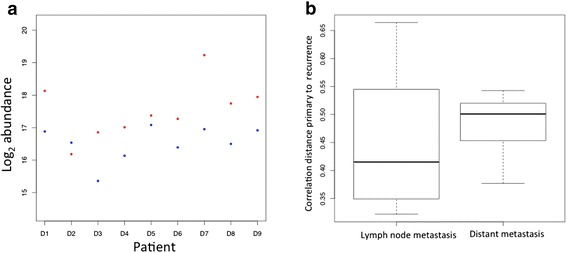
A paired analysis approach using tumours from the same patient was used. **(a)** An example of the paired analysis showing the protein levels. Log2 abundance of P16284, platelet endothelial cell adhesion molecule, for primary tumour (blue dots) and distant metastasis (red dots). **(b)**. A box plot representation of the correlation distance between paired tumours; primary-lymph node metastasis and primary-distant metastasis.

The effect of pairing samples using the example of one protein, platelet endothelial cell adhesion molecule is shown in Figure 2(a), and how it varies between primary (blue dots) and distant metastases (red dots) and between patients. If a paired analysis would not have been used the effect of the change in expression level would have been masked. In this study, we had access to paired primary—lymph node metastases and primary-distant metastases, but it is thought that this is a continuous evolution. Thus, we hypothesized that the difference between primary-lymph node should be smaller than the difference between primary-distant. Figure 2(b) shows a box plot representation of the difference between paired tumours for both primary—lymph node and primary-distant paired tumours, as correlation distance. The larger distance in the distant recurrence group, although not statistically significant corresponds to a larger difference between primary-distant tumours.

### Differences in glycoprotein expression between the primary tumour and metastases

**Figure 3:**
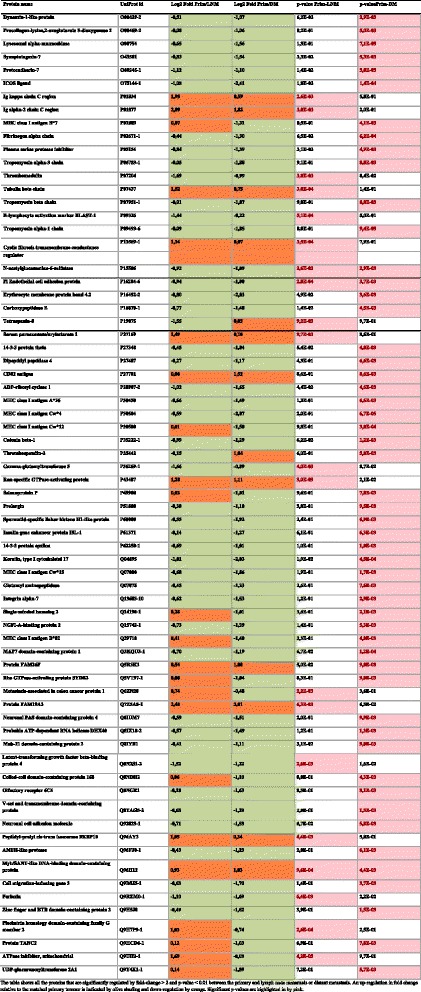
Protein expression changes between the primary tumour and lymph node metastases and distant metastases.

**Figure 4:**
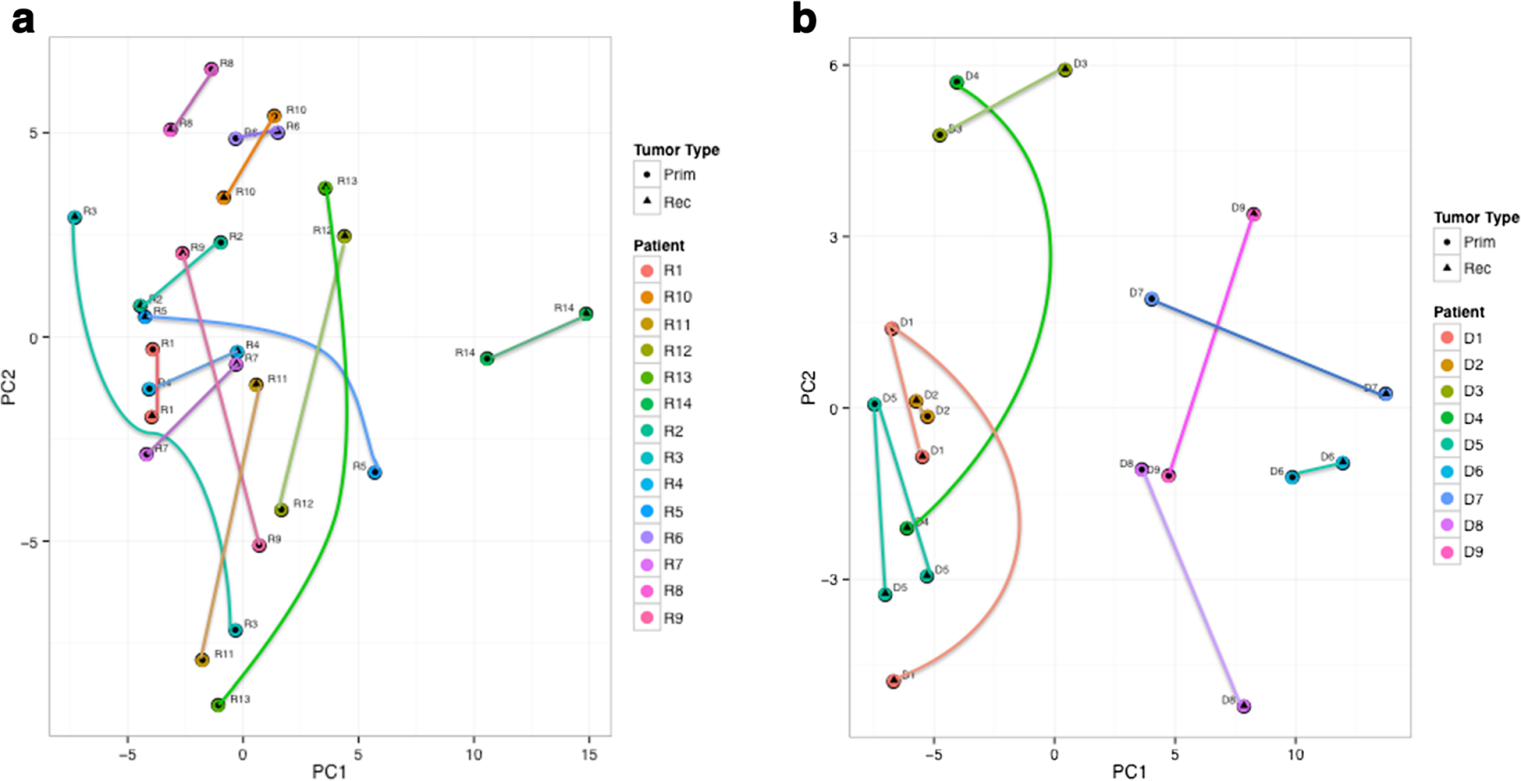
Principal component analysis shows the degree of relationship between the primary tumour and the corresponding paired metastasis. **(a)**. Principal Component Analysis of primary and lymph node metastasis pairs. A circle indicates a primary tumour and a triangle the lymph node metastasis. **(b)**. Principal Component Analysis of primary and distant metastasis pairs. A circle indicates a primary tumour and a triangle the distant metastasis.

**Figure 5:**
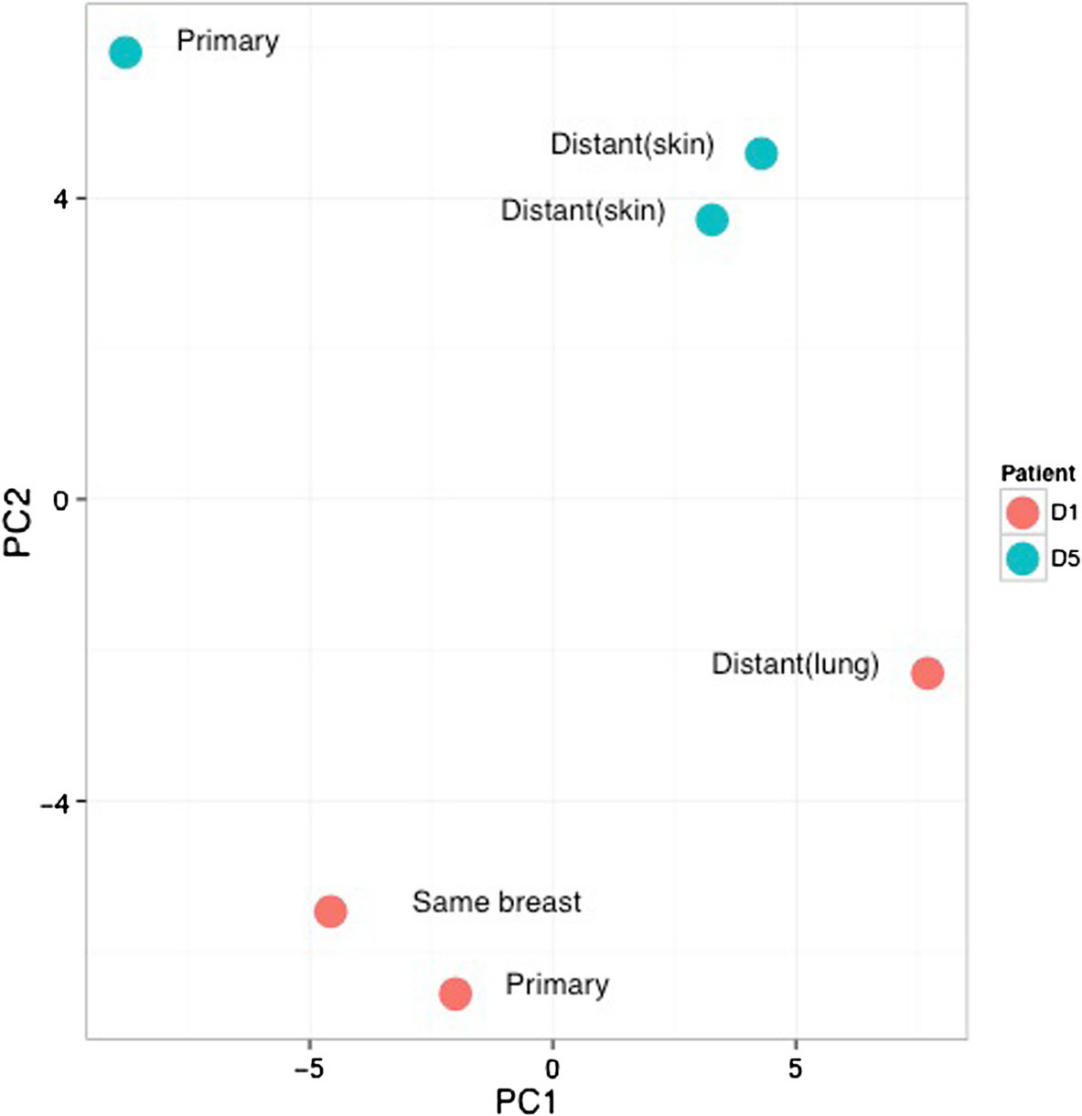
Principal Component Analysis of primary, lymph node and distant metastasis within two patients with triple samples. Red indicates patient 1 with one distant metastasis in the lung and one local recurrence and blue patient 5 with two skin metastases.

After raw mass spectra were aligned and peptide sequences identified, a Mascot search against the Uniprot database (release 2012, filtered for Human) identified over 2300 glycoproteins with a FDR < 0.05. Normalisation by total ion current in the analysis area and quantification was carried out in Progenesis. DM and LNM groups were analysed separately and Figure 3 shows proteins that are significantly regulated between primary tumour and metastases, by fold change >2 and statistical significance (p < 0.01). Further analysis of the changes occurring between glycoprotein expression in the primary tumour and the lymph node metastases is shown in Figure 4(a). Principal component analysis (PCA) of the changes occurring in glycoprotein expression between primary and LNM pairs was performed to show patterns of similarity between tumours. There is a weak tendency for the primary tumours to group together in the lower half of the figure with the lymph node metastases occurring in the top half. However there is large inter-patient variation, which is the dominant effect. The same effect is seen in the PCA plot in Figure 4(b), showing DM tumour pairs. Here the effect is even more pronounced where tumour pairs are relatively close and no obvious clustering for stage or tissue/relapse site. This is in line with the idea that breast cancer is a very heterogeneous disease and that changes between a primary tumour and the metastases is peculiar to each tumour pair, supporting the rationale for analysing tumours pairwise. It also demonstrates that the tumours do not cluster on tissue, which is a risk when analysing tumours from different sites in the body. Figure 5 shows the two patients that have three samples. Patient 1 (red) has one recurrence in the skin in the ipsilateral breast, and one in the lung, while patient 5 (blue) have two recurrences in the skin. This figure demonstrates that there is a larger difference between the two patients than between different stages of cancer (primary tumour—distant metastases). It also demonstrates that the two skin metastases from patient 5 are very similar, while the two recurrences from patient 1 are more different. This could be explained by that different changes are required for the ability to metastasize to specific sites. Possibly it is the same clone that has metastasized from patient 5 and two different clones from patient 1.

Differences in glycoprotein expression patterns between the primary tumour and metastasis could possibly be modified by any treatment given between the two samples. In this study, several patients received adjuvant treatment (Table 1). However, there were too few patients to study the effect of different treatments and we thus focused on the overall change of glycoproteins. In future studies, it would be of great interest to focus on the effect of specific treatments.

### Functional and pathway differences between the primary tumour and metastases

Among the differentially regulated proteins, there are clear groupings of proteins, for example those that are involved in the progression from primary tumour to lymph metastasis and distant metastasis, include many with motility functions. Cell migration-inducing gene 3 is up-regulated and is known to function as a cell migration-inducing gene 3 protein and is found up regulated in lung cancer as well Uniprot (Q9BSJ5-1). Protocadherin is involved in homophilic cell adhesion that allows tumour cells to move out into the blood and migrate to bone [22] as is the neuronal cell adhesion molecule (NCAM) [23] and both are up-regulated in lymph and distant metastasis. Platelet endothelial cell adhesion molecule (PECAM) also binds cells together but in addition is required for promoting the migration of leucocyte migration across the endothelium [24]. Metastasis-associated in colon cancer protein is strongly down regulated in lymph node metastasis but up regulated in distant metastases. It promotes cell motility, proliferation and hepatocyte growth factor (HGF)-dependent scattering in vitro and tumour growth and metastasis i*n vivo* (Uniprot Q6ZN28). This suggests that targeted methods could be developed to prevent dispersion at an early stage.

The proteins showing significant down-regulation such as Serum paraoxonase/arylesterase 1 (PON1) has been associated with increased vascularisation in retinopathy [25] as well as increased risk for prostate cancer [26] but in breast cancer down-regulation is associated with lower survival time in patients with clinical recurrence of breast cancer [27]. Down regulation is seen for many proteins involved in immune response such as CD82 antigen, Ig alpha-2 chain C and Ig kappa chain C though the MHC antigens class I antigen A*26, Cw*4 and Cw*15 are up-regulated.

### Changes in hormone receptor signalling pathways

Oestrogen receptor alpha (ER) is a nuclear receptor for the steroid hormone oestrogen. It is one of the clinically most important determinants of prognosis and response to endocrine treatment. The presence or absence of this receptor has a profound effect on the biology of the cancer cell and at the RNA-level, it is one of the strongest separators for clustering [28]. We therefore wanted to analyse the differences of glycoprotein expression between ER+ and ER- tumours.

Using clinical routine methods one of the patients (D3) reversed the ER status, which had an ER+ primary tumour and an ER- DM. This phenomenon has been investigated by immunohistochemistry [29] showing that 33% of patients with breast cancer show a reversal of ER or PgR status and 15% of patients experience a change in HER2 status between primary tumour and recurrence. Of clinical relevance is that ER+ patients at relapse can be treated with endocrine therapy and have better prognosis than ER- patients, independent of the ER status of the primary tumour.

Metacore analysis showed there were distinct changes occurring in hormone signalling. The program displays a thermometer indicating the change in expression of the protein in the lymph node metastasis or distant metastasis relative the paired primary tumour. One pathway that scored high and is of great clinical importance is the development of oestrogen receptor ligand independent activation. For the analysis, the protein expression was normalised against the whole primary tumour data set. Additional file 1: Figure S1(a) shows the changes in expression of the main actors in ligand independent activation (EGFR (Epidermal growth factor receptor, HER2), ErbB2 (Receptor tyrosine-protein kinase erbB-2) ErbB3 (Receptor tyrosine-protein kinase erbB-3), IGFR1 (insulin-like growth factor receptor-1), Shc (Shc transforming protein-1), NCOA2 (nuclear receptor co-activator-2), TFF1 (Trefoil factor 1) and PKA-reg (protein kinase A regulatory subunit)) in a comparison between ER+ and ER- lymph node metastasis, relative to the primary tumour. The protein Trefoil factor 1 is under control of the oestrogen receptor and is up regulated in the ER+ lymph node metastasis but clearly down-regulated in the ER- lymph node metastasis. However this is reversed in the distant metastases as seen in Additional file 1: Figure S1(b) probably as a result of the strong up-regulation of TFF1 in the ER- and of the down-regulation of the nuclear receptor co-activator-2. The effect seems to develop during the evolution of ER- lymph node metastasis to distant metastasis as seen in Additional file 1: Figure S1 (c). In contrast, in Additional file 1: Figure S1(d) no effect is seen in the change from ER+ lymph node metastasis to distant metastasis since oestrogen signalling is still intact.

### Immunohistochemical verification of mass spectrometry results

**Figure 6:**
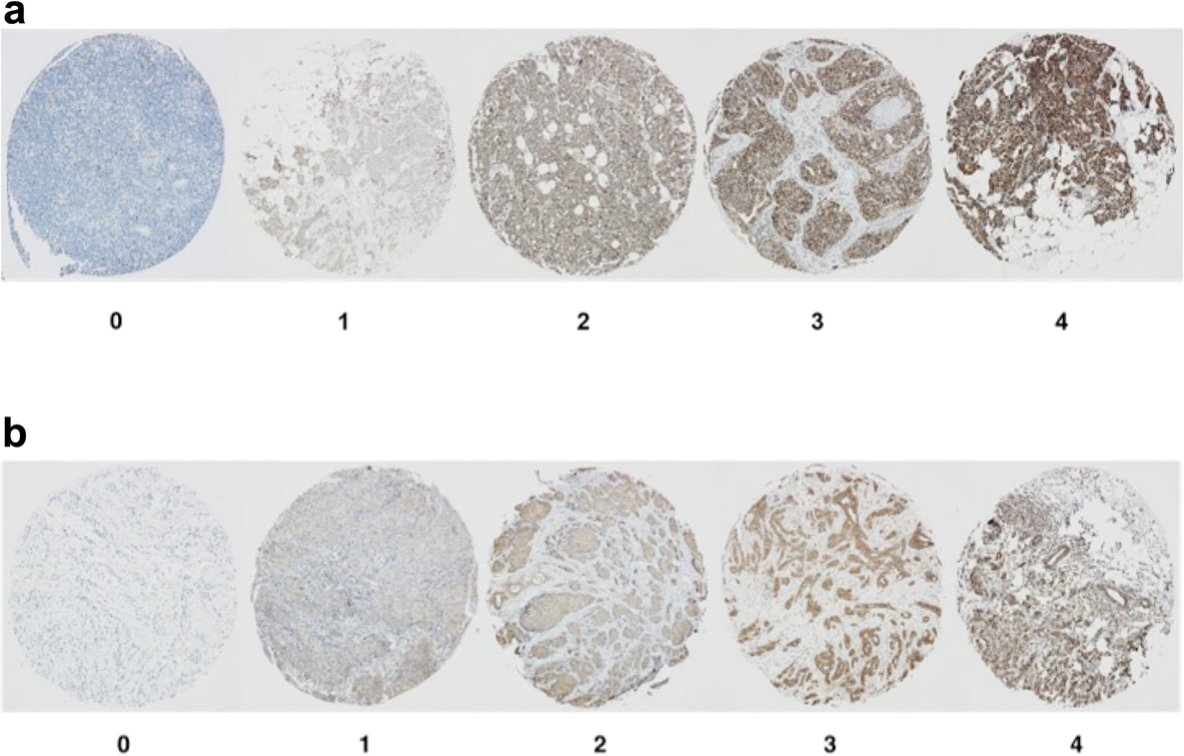
This show representative images of intensity grading levels. Tissue cores are 1.0 mm and the images were taken with 40× magnification. **a)** Staining of ATPase Inhibitory Factor 1 levels 0–4 **b)** IHC staining of tubulin β-chain intensity levels 0–4.

**Figure 7:**
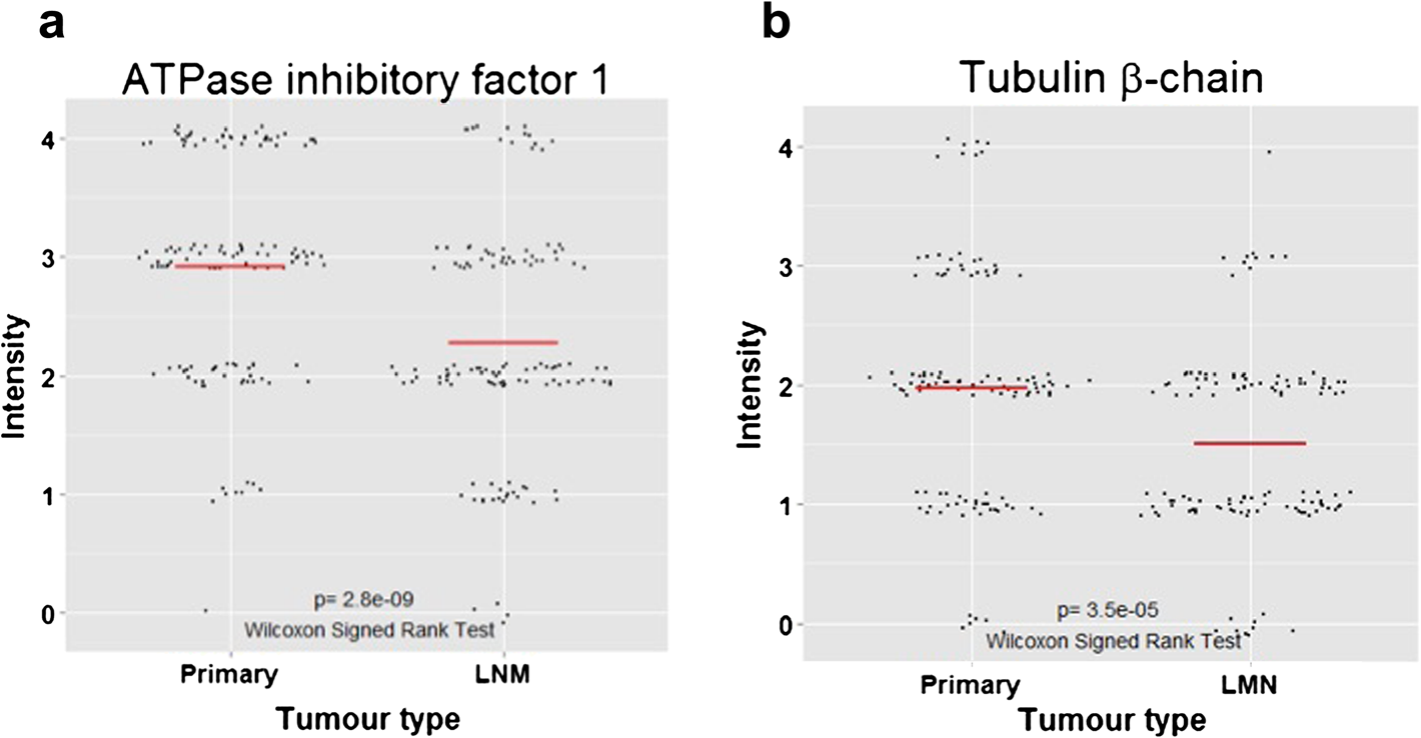
This shows the change in immunohistochemistry intensity between primary tumours and lymph node metastasis. Wilcoxon signed-rank test for paired samples were performed to assess statistical significance. Red line represents the mean. **a)** The expression of ATPase inhibitory factor 1 significantly decrease (p = 2.8e^−09^). **b)** The expression of tubulin β-chain significantly decreases (p = 3.5e^−05^).

In order to verify the accuracy of the mass spectrometry results, five of the top regulated proteins were chosen for further validation with immunohistochemistry (IHC): mitochondrial ATPase inhibitory factor 1 (ATPIF1), CD82, keratin type I cytoskeletal 17 (CK17), thrombomodulin and tubulin β-chain. Proteins were selected based on antibody availability from companies that usually provide high-quality anti-bodies. The expression was scored in 5 levels (0–4) on a tissue micro array (TMA) from a separate cohort of 552 primary tumours, 159 matched synchronous lymph node metastases and 51 matched asynchronous metastases (locoregional or distant). Patient characteristics are found in the original study [30,31]. The distribution of the intensities for the five proteins can be seen in Table 2. Representative staining intensities are shown in (Figure 6 a-b). For ATPIF1, we were able to score 126 tumour pairs, which showed a significant decrease in intensity from the primary tumours to the lymph node metastases (p = 2.8e-09) (Figure 7a). This was consistent with the down regulation found with mass spectrometry. For tubulin β-chain, we were able to score 121 tumour pairs, which showed a decrease in expression intensity from primary tumour to lymph node metastases (p = 3.5e-05) (Figure 7b). This is also consistent with the mass spectrometry observed down regulation. The IHC staining intensity was very low of the remaining antibodies; (thrombomodulin, CK17 and CD82) and we found that it was not possible to compare primary tumours and metastases (Table 2), as a large majority scored 0 intensity. This was despite great effort to optimize the IHC protocol. Thus, the IHC analysis was able to verify the decrease of ATPIF1 and tubulin β-chain expression seen with mass spectrometry, while CD82, CK17 and thrombomodulin analyses were excluded due to poor staining.

**Table 2:** **Immunohistochemical staining intensities**

**Intensity**	**ATPIF1**	**Tubulin β-chain**	**CD82**	**CK17**	**Thrombomodulin**
Primary tumour	126	121	121	122	117
0	1	6	96	144	89
1	10	29	23	5	23
2	34	56	2	2	4
3	49	22	0	0	1
4	32	8	0	1	0
Synchronous lymph node metastasis	126	121	121	122	117
0	3	8	85	116	91
1	21	54	33	2	24
2	55	49	3	3	1
3	33	9	0	1	1
4	14	1	0	0	0
Asynchronous metastasis			32	35	
0			17	31	
1			9	2	
2			3	2	
3			2	0	
4			1	0

ATPIF1 is the inhibitor of the ATP synthase, which provides the majority of oxygen necessary for sustained cellular activity [32]. Previously, it has been shown that ATPIF1 is overexpressed in human carcinomas, blocking the ATP synthase and decreasing the ATP production. This stimulates aerobic glycolysis, which in cancer cells is known as the Warburg phenotype [33,34]. Such reprogramming of the cellular energy metabolism is considered to be an emerging hallmark of cancer. Although it is known that ATPIF1 is overexpressed in several carcinomas, it is more difficult to find studies investigating and comparing the expression of ATPIF1 in primary tumours and lymph node and distant metastases [35]. Although the Warburg phenotype has been shown to stimulate metastasis, we see a decrease in ATPIF1 expression from primary tumour to lymph node metastasis. One explanation could be that the expression of ATPIF1 levels out after induction of metastasis, and only increases in expression during the transformation process from normal cell to tumour cell.

Tubulin β-chain builds up heterodimers of tubulin, which in turn are the major components of microtubules. Microtubules play a very important role in formation of the mitotic spindle, which separates the chromosomes during cell division. Furthermore, microtubules are crucial constituents of the cytoskeleton, making them important facilitators of transport and cell motility [36]. This also makes them good targets for cancer therapy, so called anti-mitotics, which bind to β-tubulin and disrupt the microtubules in the mitotic spindle. This prevents separation of the sister chromatids and hence halts mitosis [37]. The reason for a decreased level of β-tubulin in lymph node metastases is not obvious and needs to be further studied. However, here it is found in two separate patient cohorts, and with two independent techniques.

## Conclusions

In this study we compared differential glycoprotein expression in a unique set of matched tumour pairs, 14 lymph node metastases and 9 distant metastases. We used a paired approach and showed a large difference in glycoprotein expression between the primary tumour and its matched metastases, as well as large interpersonal differences.

Further, the glycoproteins here found to be differentially regulated gives an important insight into the evolution of a primary tumour towards the spreading of the disease as seen by changes in cell adhesion and migration pathways and immune response. We also demonstrate the glycoprotein difference between ER+ and ER- and that ER ligand independent activation seems to be an important pathway. Finally, we validated the differential expression of two glycoproteins: ATPIF1 and β-tubulin which confirms the validity of the mass spectrometry results and that loss of ATPIF1 and β-tubulin may be important events for a breast cancer to be able to metastasize.

In summary, there are substantial changes at the glycoprotein level during tumour development indicating that future research should be aimed at the biology of the metastasis to be able to tailor treatment according to the characteristics of the metastases, since treatment strategies are normally based on the primary tumour.

## Methods

### Materials

All HPLC solvents, Ammonium bicarbonate, 2,2,2-Trifluoroethanol (TFE), Dithiothreitol (DTT), N-acetyl-cysteine and iodoacetamide (IAA) were purchased from Sigma Aldrich (Stockholm, Sweden). Criterion Precast SDS-PAGE gels (12.5%) and Affi-Prep Hydrazide Hz resin supplied as 50% slurry in isopropanol were from BioRad (Hercules, CA, USA). Protein Desalting Spin Columns and Sodium meta-periodate were acquired from Pierce (SDS diagnostics, Falkenberg, Sweden). UltraMicroSpin C18 and UltraMacroSpin C18 columns were from the NestGroup (Southborough, MA, USA). Sequencing grade modified Trypsin was purchased from Promega (Madison, WI, USA), Zirconia beads from BioSpec Products, Inc. (Bartlesville, OK, USA). RapiGest and Sep-Pak C18 from Waters (Milford, MA, USA). PNGase F and Protease Inhibitor cocktail were acquired from Roche Gmbh (Mannheim, Germany).

### Patient data

Frozen samples from 23 patients were retrieved from the bio bank of the Department of Oncology, Lund University (Table 1). The criteria for selection were to have both a primary breast tumour sample and a sample from a lymph node metastases or distant metastases stored in the bio bank. We divided the patients into two groups, based on the type of metastasis. The first group had lymph node metastasis (LNM) (i.e. in the in the ipsilateral axilla) and the second group had distant metastasis (DM) (i.e. at any other site than the same breast and the ipsilateral axilla). We further divided the DM group based on site of the recurrence: abdomen (usually the greater oment), lung, skeleton, skin, contralateral axilla and ipsilateral breast. Two patients had 3 samples, one with a primary tumour and two distant metastases in the skin, and one with a primary tumour and metastasis to the lung as well as a recurrence in the same breast (local recurrence). The patient with a local recurrence was included in the DM group since this patient also had a lung metastasis. The local recurrence was included in the PCA plot for visualisation but was excluded from the comparison between primary tumour and distant metastasis.

The patients underwent primary surgery, either mastectomy or breast conserving surgery, in the South Sweden health care region between the years 1987–2000. They obtained standard of care after primary surgery, which for the DM group means between the primary tumour and distant metastasis. The treatment given was adjuvant radiotherapy (DM: 22%, LNM: 78%), adjuvant endocrine therapy (Tamoxifen, aromatase inhibitor, oophorectomy or radio castration) (DM: 22%, LNM: 64%) and adjuvant chemotherapy (FEC or CMF) (DM: 11%, LNM: 43%). Two patients in the LMN group received neoadjuvant chemotherapy (FEC). For patients with lymph node metastasis, the axillary surgery was performed either at the same time as primary surgery, or in the following 2–3 weeks, after cancer cells was detected in sentinel node, or other pathologic indication for additional surgery. Patients with distant metastasis were operated for the second time 0–11 years after the primary surgery. In one case, a skin metastasis was found and removed before performing mastectomy. The indication for secondary surgery and removing a distant metastasis could be a pathologic fracture, an unknown skin lesion or to obtain diagnosis for an unknown tumour.

Tumour samples were directly put on ice during surgery and stored in −80°C until analysis. A slide was taken from each tumour piece to be analysed, both primary and metastasis, and stained with haematoxylin and eosin and verified to have cancer tissue present. Follow-up and patient data was obtained through examination of patient records and from tumour analysis performed at the bio bank. The study was approved by the ethical committee of Lund University (LU 240–01).

### Clinico-pathological variables

Oestrogen receptor (ER) status was obtained from routine pathologic examination with immunohistochemistry (IHC) with a cut off of 10% or from an enzyme immuno-assay with a cut off of 25 fmol/mg protein.

### Sample preparation

Frozen tissue samples were pulverised in bead beater 4 × 1 min at the highest speed and lysed with 0.5 ml lysis buffer (50% 2,2,2-Trifluoroethanol (TFE), 50% PBS). RapiGest, 1% in 100 mM ammonium bicarbonate was added to the samples and the lysates were centrifuged at 2000 g for 30 min, at 4°C, after which supernatant was incubated at 60°C, with shaking for 2 hours. Samples were then centrifuged at 2000 g for 10 min, at RT and supernatant was reduced and alkylated by incubation with 5 mM DTT at 60°C, 800 rpm for 30 min followed by incubation with 25 mM IAA at RT, in dark for 30 min. Samples were digested with 7.9 mg/ml Trypsin at 37°C, 1000 rpm, overnight. The digest was submitted to reverse-phase clean up in 500 mg C_18_ cartridge. The peptides were eluted from the cartridge with 0.1% FA in 50% ACN and the flow-through was concentrated in a SpeedVac, to 100 μl final volume.

### Sample modification and digestion

Samples were diluted with oxidation buffer and sodium meta-periodate in water was added to the sample to a 8 mM concentration and samples incubated at 6°C, dark at 600 rpm for exactly 60 minutes. Reverse-phase clean up of digest was performed again and samples were concentrated in SpeedVac to about 400 μl. 500 μl of the supplied 50% slurry in isopropanol was washed with 3x500 μl of water; 3x500 μl of coupling buffer and after which the resins were diluted with coupling buffer to a 50%-slurry. The concentrated samples were resuspended with 400 μl coupling buffer and pH adjusted to 4.5 and then 500 μl of the 50%-slurry in coupling buffer was added to each sample and incubated at head-over rotation, RT overnight. Supernatant was collected by centrifugation (non-bound peptides) and resin washed with 2×500 μl of coupling buffer and collected into the same tube (glycopeptides).

Samples were washed with 5×500 μl each of 5 M sodium chloride, HPLC-H2O, 80% ACN, Methanol, HPLC-H2O and 100 mM ammonium bicarbonate. The resins were transferred to a new Eppendorff tube with 300 μl 100 mM Ammonium bicarbonate buffer, pH 8.0. PNGase F (5 μl of a 1U/μl) was added to the resin, pH adjusted to 8.0 and resins incubated at 37°C. After the incubation, pH was adjusted to 8.0, samples centrifuged at briefly in a mini-Eppendorf centrifuge for 30 sec and these formerly N-glycosylated peptides, collected by pipetting into a new tube. The resins were washed 2× with 500 μl 80% ACN, and flow-through combined with the previous one. Samples were then dried in SpeedVac, to volume of 100 μl. Reverse-phase clean up of digest was performed again, peptides eluted with 0.5 ml 0.1% FA/50% ACN into a new tube and then dried completely in Speed-Vac. Dried samples were resuspended with 20 μl of 0.1%FA/0.3% ACN in HPLC-grade water.

### Mass spectrometry analysis

An ESI-LTQ-Orbitrap XL mass spectrometer (Thermo Electron, Bremen, Germany) interfaced with an Eksigent nanoLC plus HPLC system (Eksigent technologies, Dublin, CA, USA) was used for all analyses. Peptides were loaded a constant flow rate of 10 μl/min onto a pre-column (PepMap 100, C18, 5 μm, 5 mm × 0.3 mm, LC Packings, Amsterdam, Netherlands) and subsequently separated on a 10 μm fused silica emitter, 75 μm × 16 cm (PicoTip™ Emitter, New Objective, Inc. Woburn, MA, USA), packed in-house with Reprosil-Pur C18-AQ resin (3 μm Dr. Maisch GmbH, Ammerbuch-Entringen, Germany). Peptides were eluted with a 150 min for label-free quantification linear gradient of 3 to 35% acetonitrile in water, containing 0.1% formic acid, with a flow rate of 300 nl/min.

The LTQ-Orbitrap was operated in a data-dependent mode simultaneously acquiring MS spectra in the Orbitrap (from m/z 400 to 2000) and MS/MS spectra in the LTQ. Four MS/MS spectra were acquired using CID (collision induced dissociation) in the LTQ and each Orbitrap-MS scan was acquired at 60,000 FWHM nominal resolution settings using the lock mass option (m/z 445.120025) for internal calibration. The normalized collision energy was set to 35% for CID. The dynamic exclusion list was restricted to 500 entries using a repeat count of two with a repeat duration of 20 seconds and with a maximum retention period 120 seconds. Precursor ion charge state screening was enabled to select for ions with at least two charges and rejecting ions with undetermined charge state.

### Data analysis

Raw mass spectrometric data was independently analysed in Progenesis LC-MS (Nonlinear Dynamics Ltd, version 4.1.4804) and Proteios SE (version 2.19.0) software platforms. In both cases, runs were aligned and peptide identifications were propagated between runs in order to minimize missing values. MS/MS spectra of ions with charge +2/+3/+4 between 400–1000 m/z and 27–62 min. were filtered and submitted to Mascot for identification using the UniProt database release 2012 filtered for human. Identifications were filtered with an FDR of 0.05 at the peptide and protein levels. Only peptides showing an Asn to Asp conversion with a consensus sequence of NX(S/T) were used for subsequent analysis. Only proteins with at least one unique peptide were kept in the dataset. Qlucore Omics Explorer (Qlucore AB, version 2.3) software was used for statistical analysis of the protein expression profiles. The functional enrichment and pathway analysis was carried out with MetaCore™ (Thomson Reuters, version 6.14).

### Statistical analysis

Normalized protein abundances (by Total Ion Current in the area of analysis) were used for the analysis. The abundances were log2 transformed. For each sample, the average log2 abundance over two technical replicates was calculated. A pairwise t-test for each protein was made between the primary and metastasis tumours for both the lymph node and the distant groups. Patients with missing values were removed for these calculations. Histograms of the p-values were made for both the lymph node and the distant metastasis groups. The histograms have a high count at low p-values showing that there are significant differences between the primary and metastasis tumours after multiple hypothesis correction. Principal component analysis (PCA) plots were made for both the distant and lymph node tumours. For each pair of primary and metastasis tumour from the same patient, the correlation distance between the two tumours was calculated. A box plot was made for the pair distances comparing the lymph node and distant groups. The mean value of the distance between pairs shows no significant difference, but the variance is higher in the lymph node metastasis group (p-value = 0.05, F test). For IHC analysis, the Wilcoxon signed-rank test was used. All computations were done in R.

### Immunohistochemical analysis

A previously constructed tissue microarray (TMA) was used for immunohistochemistry verification. Details and patient characteristics are found in the original study [30,31]. In brief, 552 primary tumours, 159 axillary synchronous lymph node metastases and 51 asynchronous metastases (locoregional or distant), were collected. From the donor paraffin block two tissue cylinders of 1.0 mm were taken from a representative area of the tissue and transferred to a recipient paraffin block. The TMA blocks were cut into 3–4 μm thick sections and mounted on to glass slides (SuperFrost Plus, Menzel-Gläser, Gerhard Menzel GmbH, Braunschweig, Germany), the glasses were let to dry at room temperature and baked in a heat chamber at 60°C for 2 hours.

The following monoclonal antibodies were used: mouse anti-cytokeratin 17 (1:30, Dako, Glostrup, Denmark), rabbit anti-CD82 (1:500, Cell Signalling Technologies, USA) rabbit anti-β-tubulin (1:300, Cell Signalling Technologies, USA), rabbit anti-ATPIF1 (1:1500, Cell Signalling Technologies, USA) and mouse anti-thrombomodulin (1:10, Dako, Glostrup, Denmark). Slides with cytokeratin 17, CD82 and tubulin β-chain were placed in EnVision™ FLEX buffer with pH 9 (Dako, Glostrup, Denmark) heated in a PT-Link (Dako, Glostrup, Denmark) to 65°C, then 98°C for 20 min and decreased to 65°C. Slides with ATPase inhibitory factor 1 (ATPIF1) and Thrombomodulin were deparaffinised using xylene, 99% and 96% ethanol, placed in a pH 6 Target Retrieval Solution (Dako, Glostrup, Denmark). Subsequently, the slides were placed in a Pressure Cooker 2100 Retriever (Histolab Products AB, Gothenburg, Sweden) in 125°C for 5 minutes and then let to cool. Staining was performed with EnVision™ FLEX, High pH (Dako, Glostrup, Denmark) in an automatic immunohistochemistry staining machine (Dako, Glostrup, Denmark). All antibodies were incubated with the slides for 30 minutes, except the β-tubulin slides, which were incubated for 60 minutes. Counterstaining was performed using REAL™ Haematoxylin (Dako, Glostrup, Denmark) for 2 minutes. Sections were dehydrated and mounted with coverslips.

Protein levels were scored with a light microscope under the supervision of a pathologist. Cores were scored for intensity in 5 levels (0–4) and proportion as percentage of positive cells. The average between cores were calculated for each tumour and used for further analysis. An average proportion <10% were regarded as negative (intensity level 0), otherwise intensity was used for analysis. Pairs of primary and synchronous lymph node or asynchronous metastases missing data from one of the tumour types were excluded. Primary tumours and synchronous lymph node metastasis were scored. Asynchronous metastases were scored if mass spectrometry analysis showed differential regulation between primary tumour and distant metastasis.

## Additional file

Additional file 1: Figure S1. Metacore analysis showing the proteins found that are involved in the development of ligand independent oestrogen receptor activation. (a) The change in expression relative to the primary tumour is indicated by the thermometers next to the protein representation for ER+ lymph node metastases (1) and ER- lymph node metastases (2). EGFR (Epidermal growth factor receptor, HER2), ErbB2 (Receptor tyrosine-protein kinase erbB-2) ErbB3 (Receptor tyrosine-protein kinase erbB-3), IGFR1 (insulin-like growth factor receptor-1), Shc (Shc transforming protein-1), NCOA2 (nuclear receptor co-activator-2), TFF1 (Trefoil factor 1) and PKA-reg (protein kinase A regulatory subunit). (b) The change in expression, relative to the primary tumour, is indicated by the thermometers next to the protein representation for ER+ distant metastasis (1) and ER- distant metastasis (2)-. (c) The change in expression, relative to the primary tumour, is indicated by the thermometers next to the protein representation for ER- lymph node metastasis (1) and ER- distant (2) metastases. (d) The change in expression, relative to the primary tumour, is indicated by the thermometers next to the protein representation for ER+ lymph node metastasis (1) and ER+ distant (2) metastases.

## Abbreviations

LC-MS/MS: Liquid chromatography tandem mass spectrometry
ER: Oestrogen receptor alpha
DM: Distant metastasis
LNM: Lymph node metastasis
PCA: Principle component analysis
ACN: Acetonitrile
IHC: Immunohistochemistry
TMA: Tissue microarray
