# Supplementary figures and images for: Changes in glycoprotein expression between primary breast tumour and synchronous lymph node metastases or asynchronous distant metastases
Source: Clin Proteomics. 2015 May 12;12(1):13. doi: 10.1186/s12014-015-9084-7 (PMC4436114; doi:10.1186/s12014-015-9084-7)

S1(a)

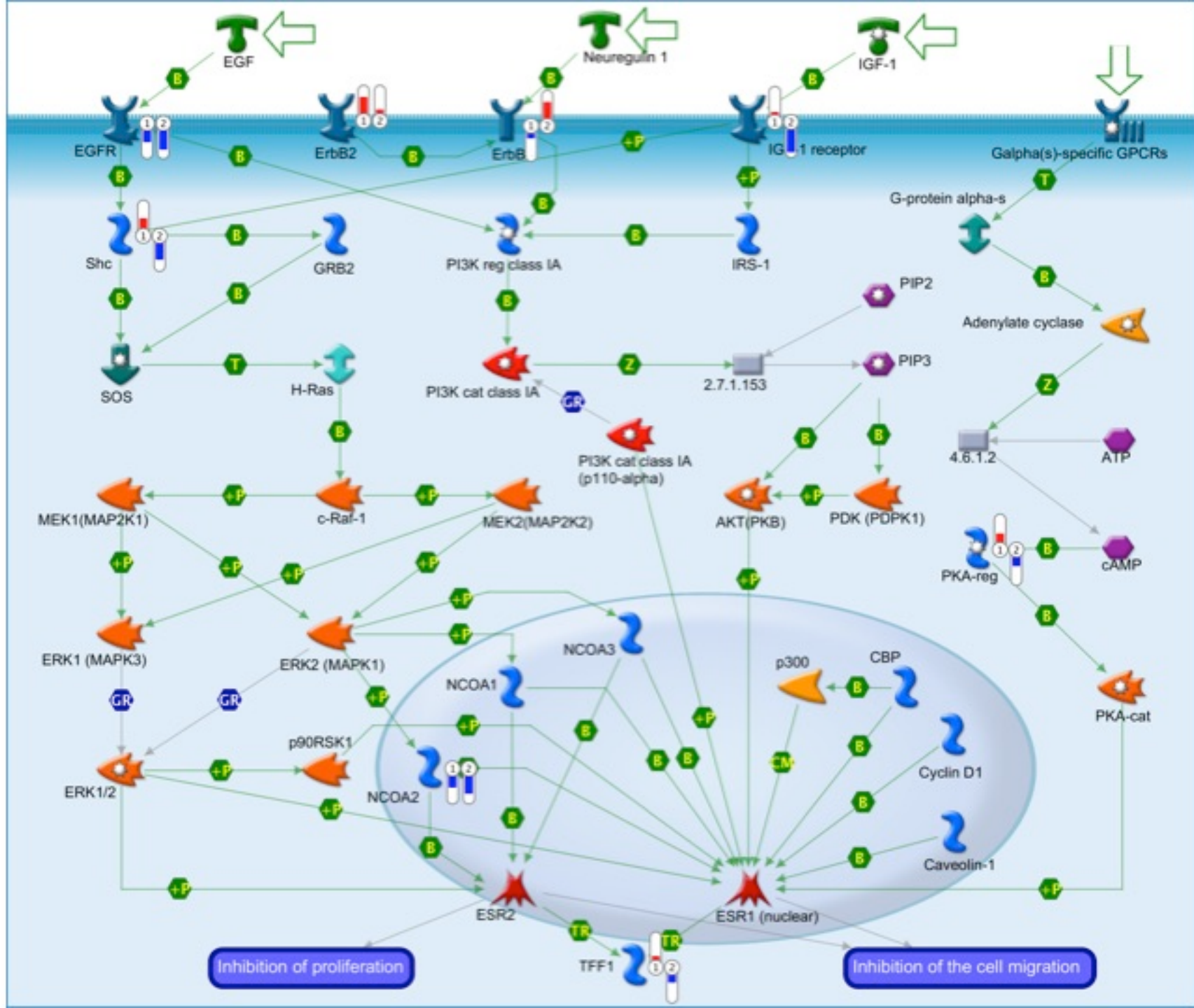

S1(b)

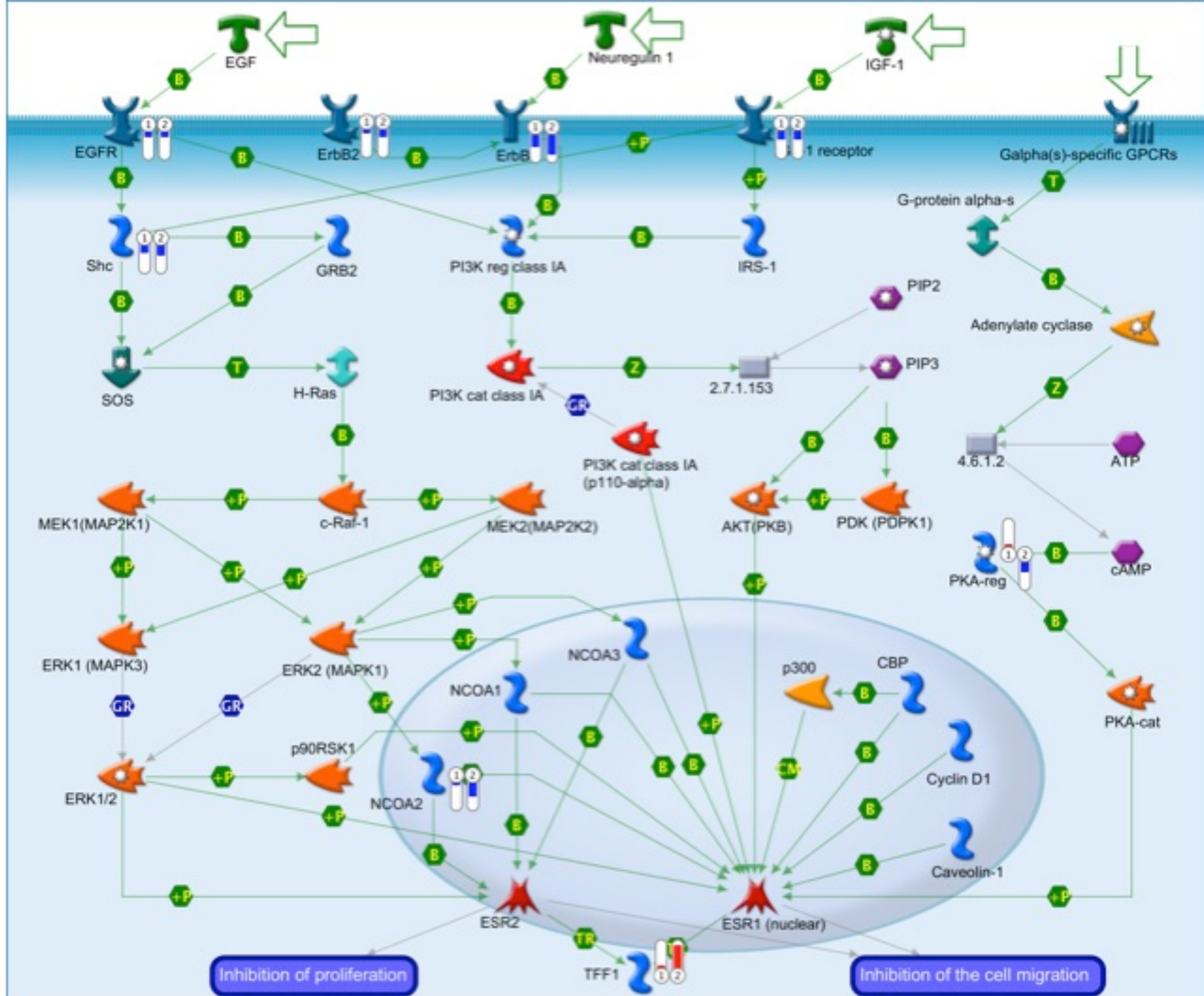

S1(c)

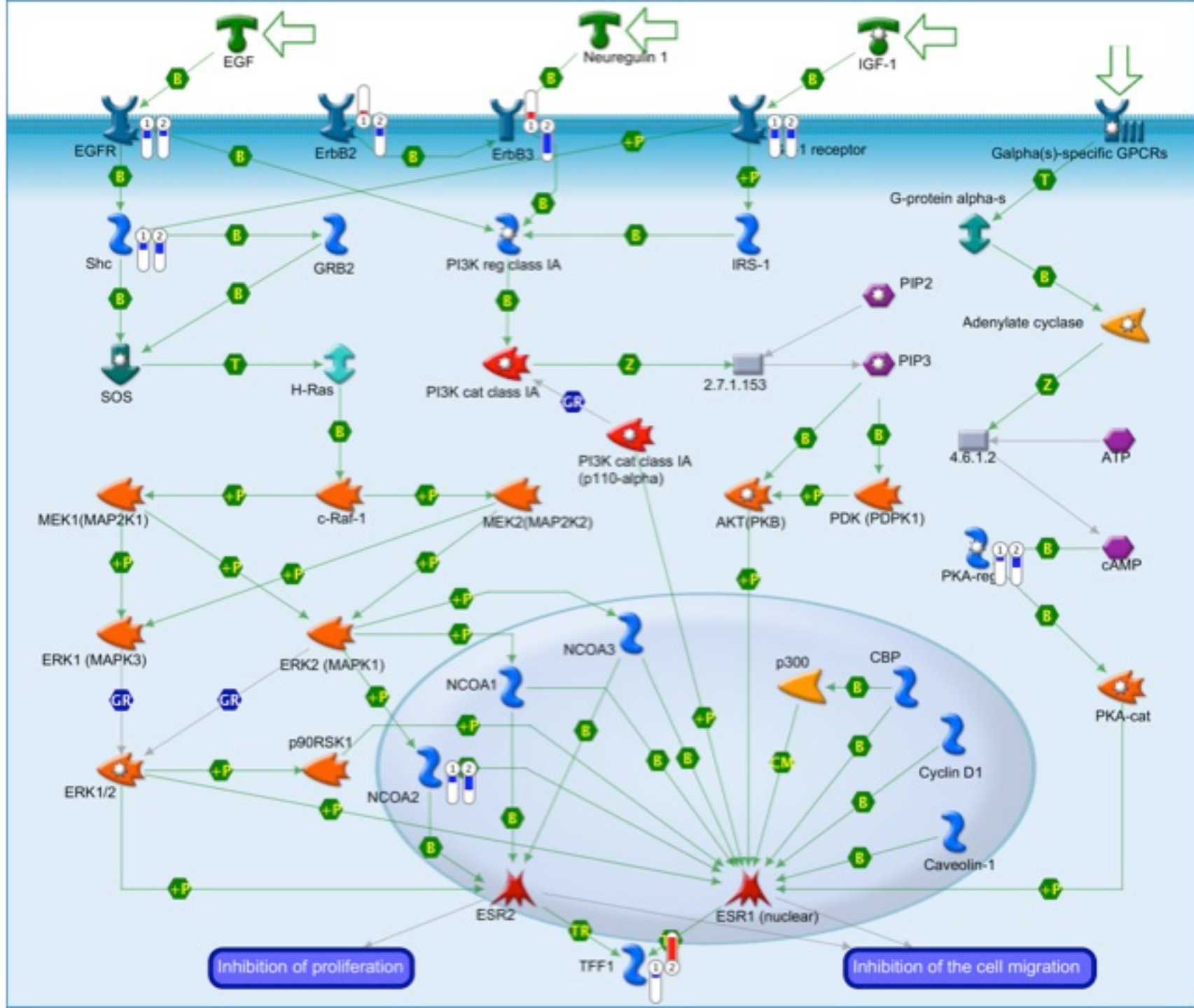

S1(d)

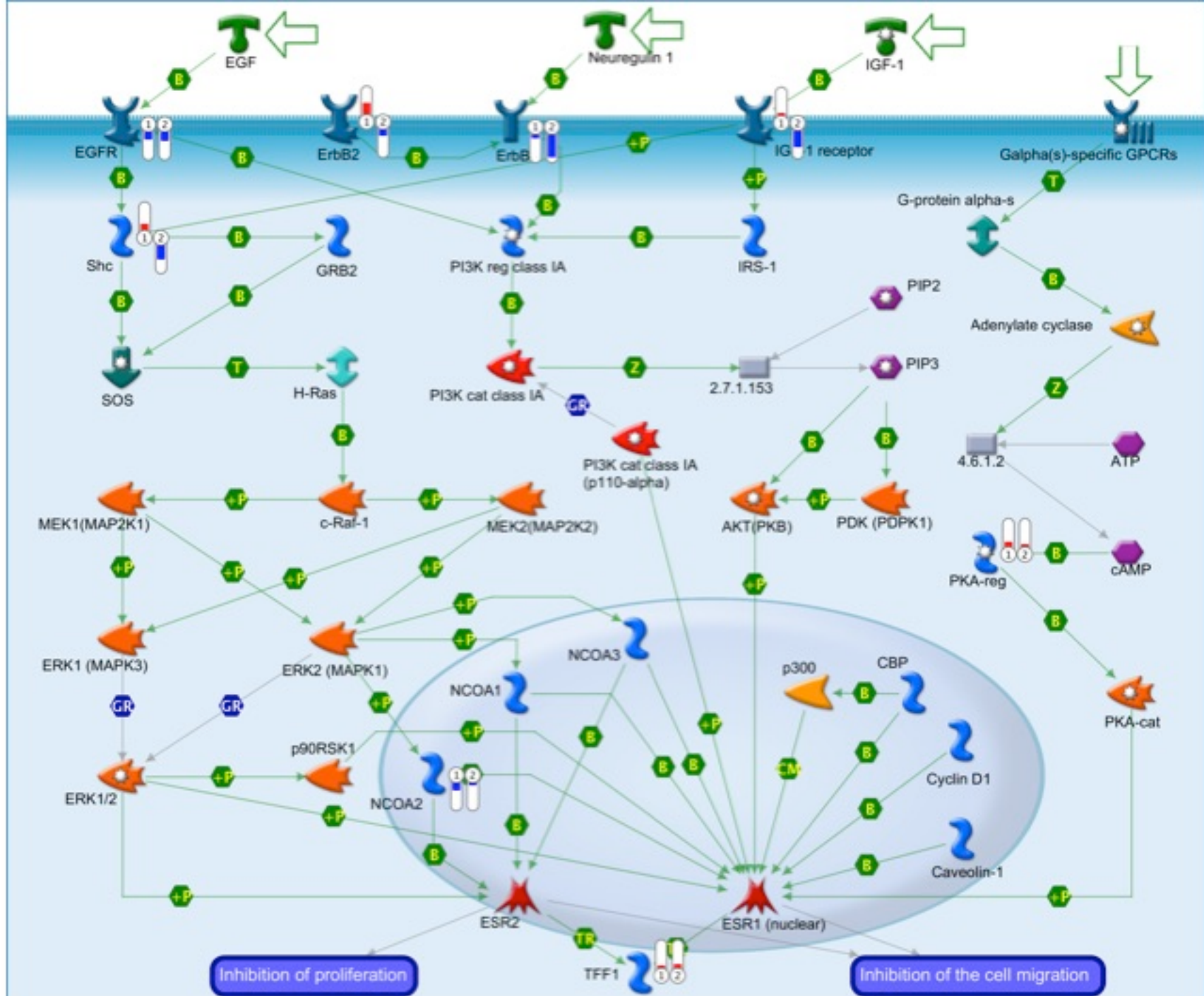

Supplement: Additional file 1: Figure S1. — Metacore analysis showing the proteins found that are involved in the development of ligand independent oestrogen receptor activation. (a) The change in expression relative to the primary tumour is indicated by the thermometers next to the protein representation for ER+ lymph node metastases (1) and ER- lymph node metastases (2). EGFR (Epidermal growth factor receptor, HER2), ErbB2 (Receptor tyrosine-protein kinase erbB-2) ErbB3 (Receptor tyrosine-protein kinase erbB-3), IGFR1 (insulin-like growth factor receptor-1), Shc (Shc transforming protein-1), NCOA2 (nuclear receptor co-activator-2), TFF1 (Trefoil factor 1) and PKA-reg (protein kinase A regulatory subunit). (b) The change in expression, relative to the primary tumour, is indicated by the thermometers next to the protein representation for ER+ distant metastasis (1) and ER- distant metastasis (2)-. (c) The change in expression, relative to the primary tumour, is indicated by the thermometers next to the protein representation for ER- lymph node metastasis (1) and ER- distant (2) metastases. (d) The change in expression, relative to the primary tumour, is indicated by the thermometers next to the protein representation for ER+ lymph node metastasis (1) and ER+ distant (2) metastases. [file 12014_2015_9084_MOESM1_ESM.pdf]
